# Hsp70-Bim interaction facilitates mitophagy by recruiting parkin and TOMM20 into a complex

**DOI:** 10.1186/s11658-023-00458-5

**Published:** 2023-05-26

**Authors:** Ting Song, Fangkui Yin, Ziqian Wang, Hong Zhang, Peng Liu, Yafei Guo, Yao Tang, Zhichao Zhang

**Affiliations:** 1 State Key Laboratory of Fine Chemicals, School of Chemical Engineering, Dalian University of Technology, Dalian, Liaoning China; 2 School of Bioengineering, Dalian University of Technology, Dalian, Liaoning China

**Keywords:** Apoptosis, Hsp70-Bim, Mitophagy, Parkin, TOMM20

## Abstract

**Background:**

For cancer therapy, the identification of both selective autophagy targets and small molecules that specifically regulate autophagy is greatly needed. Heat shock protein 70 (Hsp70) is a recently discovered BH3 receptor that forms a protein‒protein interaction (PPI) with Bcl-2-interacting mediator of cell death (Bim). Herein, a specific inhibitor of the Hsp70-Bim PPI, **S1g-2**, and its analog **S1**, which is a Bcl-2-Bim disruptor, were used as chemical tools to explore the role of Hsp70-Bim PPI in regulating mitophagy.

**Methods:**

Co-immunoprecipitation and immunofluorescence assays were used to determine protein interactions and colocalization patterns. Organelle purification and immunodetection of LC3-II/LC3-I on mitochondria, endoplasmic reticulum (ER) and Golgi were applied to identify specific types of autophagy. Cell-based and in vitro ubiquitination studies were used to study the role of the Hsp70-Bim PPI in parkin-mediated ubiquitination of outer mitochondrial membrane 20 (TOMM20).

**Results:**

We found that after the establishment of their PPI, Hsp70 and Bim form a complex with parkin and TOMM20, which in turn facilitates parkin translocation to mitochondria, TOMM20 ubiquitination and mitophagic flux independent of Bax/Bak. Moreover, **S1g-2** selectively inhibits stress-induced mitophagy without interfering with basal autophagy.

**Conclusions:**

The findings highlight the dual protective function of the Hsp70-Bim PPI in regulating both mitophagy and apoptosis. **S1g-2** is thus a newly discovered antitumor drug candidate that drives both mitophagy and cell death via apoptosis.

**Graphical Abstract:**

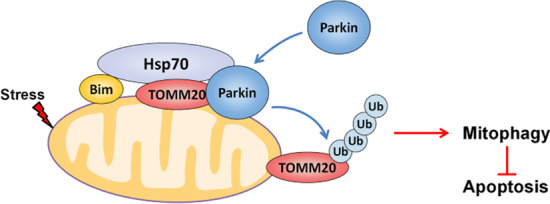

**Supplementary Information:**

The online version contains supplementary material available at 10.1186/s11658-023-00458-5.

## Introduction

Autophagy, an evolutionarily conserved mechanism, is a self-degradation process typically stimulated in cells under nutrient deprivation or cellular stress conditions [1, 2]. The basal, constitutive level of autophagy plays an important role in cellular homeostasis by eliminating damaged/old organelles and recycling long-lived proteins and protein aggregates [3]. Thus, it maintains the quality of essential cellular components. When cells encounter environmental stresses, including tumorigenic environment stress and anticancer drug treatment, the level of autophagy can be markedly increased as a cytoprotective response, resulting in cell adaptation and survival [4, 5]. Hence, recent studies have revealed that inhibition of stress-induced autophagy may be a promising therapy for cancer [6, 7].

Although autophagy has long been considered a nonselective catabolic process, various cellular components have been recently identified as cargoes in selective autophagy [8]. Mitophagy, for example, ensures the selective removal of damaged or dysfunctional mitochondria and is a flexible mechanism that supports the metabolic adaptation and survival of cancer cells within the harsh tumor microenvironment [9]. It is also crucial to cancer cell plasticity and response to cancer therapies. Therefore, understanding mitophagy factors and mechanism(s) on which tumorigenesis depends and tumor drug-resistance relies is needed for the development of novel therapeutic strategies aiming to proficiently harness mitophagy in anticancer therapy.

Parkin-mediated mitophagy has been shown to play an extensive role in tumor suppression [10]. A diverse siRNA screening strategy validated that HSPA1L (Hsp70 member) and Bag4, which regulate parkin translocation, are useful drug targets for increasing mitochondrial quality control [11]. Bim was also included among the strong candidates because siRNA directed to it affected parkin translocation to the same degree as HSPA1L [11].

Recently, our group reported that the Hsp70-Bim dimer recruits the oncogenic clients AKT, Raf-1, and eIF4E and mediates eIF2 signaling, the regulation of eIF4E and p70S6K signaling, and mTOR signaling pathway activation s to support chronic myeloid leukemia (CML) cell survival [12–14]. Interestingly, parkin and TOMM20 have been identified in the Hsp70 interactome, which is significantly affected by a specific Hsp70-Bim disruptor, **S1g-2** [12]. However, whether and how Hsp70-Bim is involved in the mitophagy pathway remain unclear. As a stress-related rheostat, Bcl-2-containing complexes involve multiple components that engage in both apoptosis and autophagy mediated through their BH3 motif [15]. Therefore, Bcl-2 family proteins are global regulators of mitochondrial homeostasis. Since Hsp70 has been demonstrated to be a BH3 receptor and to form a complex with Bim through the BH3 groove [13], it is reasonable to think that Hsp70 also regulates cell signaling, in addition to its chaperone activity.

To identify the role of the Hsp70-Bim PPI in autophagy, the effects of **S1g-2** on autophagy and that of its analog **S1** were analyzed [16–18]. **S1** has been identified as a Bcl-2-Bim disruptor through which it enhances apoptosis. Our results revealed opposite regulatory effects of **S1g-2** and **S1** on autophagy. The selective mitophagy promotion of Hsp70-Bim was found to be due to the formation of a complex involving parkin and TOMM20, which facilitates parkin-mediated TOMM20 ubiquitination. **S1g-2** selectively binds Hsp70 in situ and promotes apoptosis by inhibiting cytoprotective mitophagy, rendering it a novel mechanism by which apoptosis is enhanced.

## Materials and methods

### Reagents

The compounds VP-16 and BafA1 were purchased from Selleck Chemicals (Houston, TX, USA). All the chemicals were dissolved in dimethyl sulfoxide (DMSO) to a concentration of 10 mM. To obtain the final concentration, stock solutions were diluted in culture medium. The recombinant BimLΔC27 protein was purchased from Sino Biological Inc. (Cat: 13816-H07E, Beijing, China). Primary antibodies against LC3 (sc-398822), p62 (sc-28359), β-actin (sc-8432), Bim (sc-374358), Beclin 1 (sc-48341), Hsp70 (sc-24), and Mcl-1 (sc-74436) were purchased from Santa Cruz Biotechnology (Santa Cruz, CA, USA). Antibodies against Bcl-2 (ab32124), TOMM20 (ab283317), calnexin (ab92573), GM130 (ab32337), parkin (ab77924) and PINK1 (ab300623) were purchased from Abcam plc (Cambridge, MA, UK). Antibodies against cytochrome c (#4272) and AMPK (#2532) and specific antibodies against p-AMPK (Thr172) (#2531) were purchased from Cell Signaling Technology (Beverly, MA, USA).

### Cell lines

The HEK293T human embryonic kidney cell line and HeLa human cervical cancer cell line were purchased from American Tissue Culture Collection (ATCC) and used within 6 months of resuscitation. The cells were cultured in DMEM (Thermo Scientific HyClone, Beijing, China) supplemented with 10% fetal bovine serum (Gibco BRL, Grand Island, NY, USA) and 100 U/mL penicillin‒streptomycin (Thermo Scientific Hy Clone, 15140148). The cells were cultured in an environment with 5% CO_2_ at 37 °C in a humidified incubator (Thermo Scientific, D-63450).

### In vitro ubiquitination assay

HEK293T cells were immunoprecipitated using an anti-parkin antibody (300 μg). A 20-μL ubiquitination reaction mixture contained immunocomplexes, and 1.2 μM recombinant TOMM20 was incubated in ubiquitination reaction buffer (25 mM Tris–HCl (pH 7.5), 50 mM NaCl, 4 mM ATP, 4 mM MgCl_2_, 2 mM DTT, 10 mM phosphocreatine, 0.5 U creatine kinase, and 20 μM ZnCl_2_) in the presence of 50 ng of the human E1 enzyme, 500 ng of the Ubch7 E2 enzyme, and 10 μg of ubiquitin. Then, the mixture was subjected to a ubiquitin system with recombinant Hsp70 (1 μM) alone or in combination with Bim (0.1 μM). Reaction mixtures were incubated at 37 °C for 4 h and stopped by adding 10 μl of SDS sample buffer and heating at 95 °C for 5 min. The samples were analyzed by SDS‒PAGE and immunoblotting with an anti-TOMM20 antibody. To evaluate the influence of **S1g-2** on TOMM20 ubiquitination, 10 mM **S1g-2** was stored in 100% DMSO, diluted with DMSO and added to the reaction buffer to a final concentration of 5 μM (5% DMSO).

### Mitochondria, ER and Golgi isolation

Mitochondria were obtained using a mitochondria extraction kit for use with animal tissue according to the manufacturer’s instructions (Genmed Scientifics Inc., Shanghai, China). ER and Golgi were isolated using an endoplasmic reticulum isolation kit (Sigma, 038M4161V) and Golgi isolation kit (Sigma, GL0010) following the manufacturer’s instructions. The protein concentration was measured using a BCA kit (Pierce, Rockford, IL). The isolated organelle fraction was collected for Western blot assay.

### Plasmid construction and transfection

GFP-LC3 was obtained from Addgene (Cambridge, MA, USA) (Plasmid 21073). Full-length parkin cDNA was subcloned into a C1 vector (Addgene plasmid 54607) to generate a GFP-tagged parkin construct. GFP-LC3 and GFP-parkin were transfected into HEK293T and HeLa cells, respectively, using Lipofectamine 2000 Transfection Reagent (Thermo Fisher Scientific, Waltham, MA, USA). Negative control shRNA (shNC), parkin shRNA, Hsp70 shRNA and Bim shRNA were purchased from Santa Cruz Biotechnology. The viral particles were combined with 8 μg/mL polybrene and used to infect HEK293T cells overnight. The cell culture medium was replaced with fresh complete growth medium, and after 48 h, the cells were used for experiments.

### Cell imaging

Monolayer cells were visualized with an Olympus FV-1000 inverted fluorescence microscope. GFP-LC3 was used to label autophagosomes, and MitoTracker Red CMXRos (Molecular Probes, Eugene, OR, USA) was used to label mitochondria. The images were scanned through a plane with reference to the middle of a sphere. The acquired images were exported to Adobe Photoshop, processed and then imported into ImageJ for generating RGB split images and colocalization analysis with a colocalization RGB plugin.

### In situ proteome labeling and pull-down assays

HEK293T cells were cotreated with HBSS and **S1-probe**, **S1g-2-probe** or **S1n-probe** for 4 h. Then, the medium was aspirated, and the cells were washed gently with PBS (2 ×) to remove excess probe and then subjected to UV irradiation for 20 min on ice. The cells were trypsinized and pelleted by centrifugation. Then, the cell pellets were resuspended in PBS (50 μL) and homogenized by sonication, and the cell solution was diluted to 1 mg/mL with PBS. One microliter of a freshly premixed click chemistry reaction cocktail in PBS (2.5 mM Biotin-N3 from the 10 mM stock solution in DMSO, 2.5 mM TBTA from a 10 mM concentration of freshly prepared stock solution in deionized water, 25 mM TCEP from a 100 mM concentration of freshly prepared stock solution in deionized water, and 25 mM CuSO4 from a 100 mM concentration of freshly prepared stock solution in deionized water) was added. The reaction mixture was further incubated for 2 h with gentle mixing before being terminated by the addition of prechilled acetone (0.5 mL; 30 min incubation at − 20 °C). The precipitated proteins were subsequently collected by centrifugation (13,000 rpm × 10 min at 4 °C), resolubilized in 1% SDS in 50 μL of PBS and then incubated with avidin − agarose beads (100 μL/mg of protein) at 4 °C overnight. After centrifugation, the supernatant was removed. The beads were washed with 0.1% SDS once and PBS four times and then boiled in SDS loading buffer (2 ×) [200 mM Tris, 400 mM dithiothreitol (DTT), and 8% SDS, pH 6.8] for 15 min. The proteins thus obtained were separated by SDS − PAGE (10% gel) and then visualized by silver staining.

### Statistical analysis

Statistical analysis was performed using IBM SPSS software (version 20.0, IBM, Armonk, NY), and all data are presented as the mean ± SD. Significant differences for more than 2 groups were analyzed by one-way ANOVA. The analysis of the time course experiments shown in Fig. 6C and Additional file 1: Fig. S8 was performed by two-way ANOVA. A value of *P* < 0.05 was considered to be significant.

## Results

### A pair of small molecules that disrupt Hsp70-Bim and Bcl-2-Bim affect autophagy in opposite ways

**Fig. 1:**
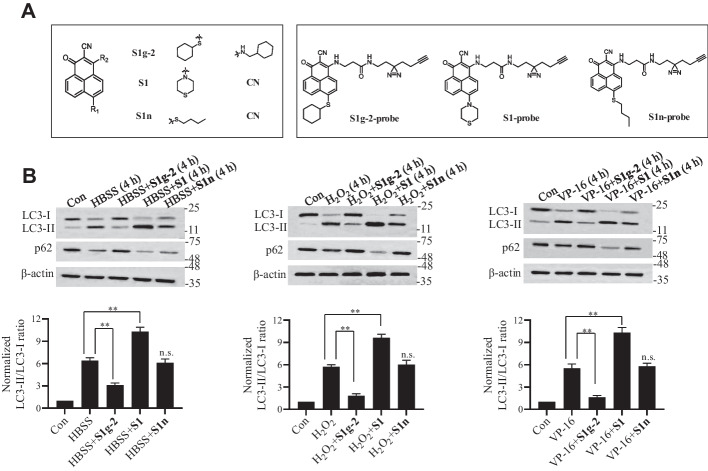
**S1g-2** and **S1** modulate a variety of stress-induced autophagy in opposite ways.** A** Chemical structures of **S1g-2**, **S1**, **S1n**, **S1g-2-probe**, **S1-probe** and **S1n-probe**. **B** Western blot analysis of the levels of LC3 and p62 in HEK293T cells treated with 0.5 mM H_2_O_2_, 34 μM VP-16 or HBSS in the presence or absence of 10 μM **S1g-2**, 10 μM **S1** and 10 μM **S1n** respectively for 4 h, using β-actin as a loading control. An equivalent of DMSO were added to the compound untreated group as vehicle control. The graphs show (mean ± SD, *n* = 3 biologically independent experiments) LC3-II/LC3-I ratios of each treatment normalized to the LC3-II/LC3-I ratio of control cells. **P < 0.01 (one-way ANOVA test); n.s. indicates no significance between HBSS + **S1n** and HBSS group

Three analogs were chosen to probe the role of Hsp70-Bim in autophagy (the structure is shown in Fig. 1A). **S1** targets the Bcl-2-Bim protein‒protein interaction (PPI), while **S1g-2** specifically disrupts the Hsp70-Bim PPI. **S1n,** showing weak effects on both complexes, was used as the control. We exposed mammalian cells to oxidative stress (H_2_O_2_), nutrient starvation by culturing them in HBSS, and the therapeutic agent etoposide (VP-16) alone or in the presence of the three analogs. Autophagy was measured by quantifying the conjugation of LC3-I with phosphatidylethanolamine, which forms LC3-II, and the p62 level [19].

As shown in Fig. 1B, the stress in HEK293T cells treated with 0.5 mM H_2_O_2_ and 34 μM VP-16 or cultured in HBSS for 4 h led to an increase in the LC3-II/LC3-I ratio and a decrease in p62 level, as expected and consistent with previous reports [20, 21]. Then, we measured the LC3 and p62 levels in HEK293T cells after a 4-h treatment with HBSS, H_2_O_2_ or VP-16 in the presence of **S1**, **S1g-2** or **S1n**. As shown in Fig. 1B, 10 μM **S1** enhanced stress-induced autophagy, which is consistent with established results showing that the inhibition of Bcl-2 activity can activate autophagy. In contrast, 10 μM **S1g-2** led to a significant decrease in the stress-induced LC3-II/LC3-I ratio and an exacerbated decrease in stress-mediated p62 levels (Fig. 1B), indicating that **S1g-2** inhibited autophagy in response to various stresses. **S1n** exhibited no effect on autophagic flux and was thus used as the negative control in subsequent experiments. A negligible death rate was measured in cells in the presence of **S1**, **S1g-2** or **S1n** for 4 h (Additional file 1: Fig. S1), indicating that the effects of these compounds on autophagy are not confounded by the induction of apoptosis.

### Hsp70-Bim PPI mediates stress-induced autophagy but not basal autophagy

**Fig. 2:**
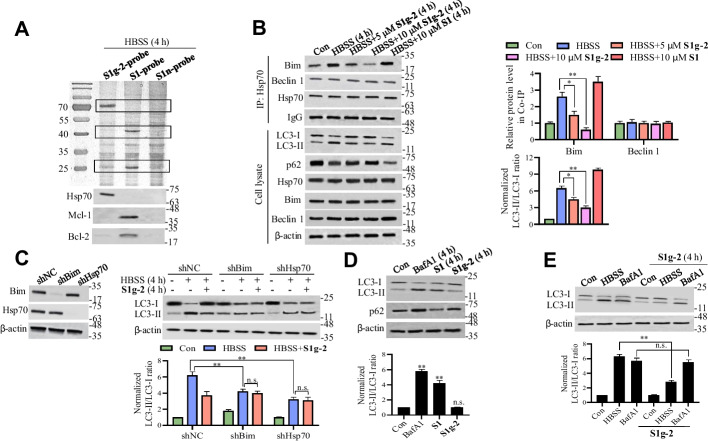
Hsp70-Bim PPI mediates stress-induced autophagy rather than basal autophagy. **A** In-gel silver staining of the proteins enriched by **S1g-2-probe**, **S1-probe** and **S1n-probe** respectively from HEK293T cells cultured in HBSS for 4 h (top); the Hsp70, Bcl-2 and Mcl-1 were further verified by Western blotting (bottom). **B** Co-IP analysis of Hsp70 interactions with Bim and Beclin 1 in HEK293T cells treated with HBSS in the presence or absence of indicated concentrations of **S1g-2** and **S1**, respectively, and Western blot analysis of LC3 and p62 levels in cell lysates. An equivalent of DMSO were added to the compound untreated group as vehicle control. The top graph shows (mean ± SD, *n* = 3 biologically independent experiments) the relative level of Bim and Beclin 1 in co-IP of each treatment normalized to that in control cells. The bottom graph shows (mean ± SD, *n* = 3) LC3-II/LC3-I ratios of each treatment normalized to the LC3-II/LC3-I ratio of control cells. *P < 0.05, **P < 0.01 (one-way ANOVA test). **C** Western blot analysis of the LC3 level in NS shRNA-transfected, Bim shRNA-transfected and Hsp70 shRNA-transfected HEK293T cells respectively with HBSS treatment in the presence or absence of 10 μM **S1g-2** for 4 h. An equivalent of DMSO were added to the compound untreated group as vehicle control. **P < 0.01 (one-way ANOVA test); n.s. indicates no significance. **D** Western blot analysis of the levels of LC3 and p62 in HEK293T cells treated with 30 nM BafA1, 10 μM **S1g-2** and 10 μM **S1** respectively for 4 h. ^**^P < 0.01 (one-way ANOVA test); n.s. indicates no significance. **E** Western blot analysis of the levels of LC3 in HEK293T cells treated with HBSS or 100 nM BafA1 in the presence or absence of 10 μM **S1g-2** for 4 h. The graphs show (mean ± SD, *n* = 3 biologically independent experiments) LC3-II/LC3-I ratios normalized to that in control cells. **P < 0.01 (one-way ANOVA test); n.s. indicates no significance

Next, we identified the autophagy-modulating targets of **S1** and **S1g-2** in the whole-cell proteome via a comparative chemical proteomic analysis. Each of the three compounds was incorporated with a well-established “minimalist” linker to generate the affinity-based probes **S1g-2-probe**, **S1-probe** and **S1n-probe** (structure shown in Fig. 1A), which were synthesized according to published methods [14]. We tested their effects on autophagy to ensure that the incorporation of the linker did not alter their bioactivities (Additional file 1: Fig. S2A). After HEK293T cells were cultured in HBSS for 4 h, which was the same as the aforementioned experimental conditions, the cells were lysates, and the lysates were incubated with the **S1g-2-probe**, **S1-probe,** or **S1n-probe**, and then, the probe-binding targets in the lysates were pulled down together with the probe via streptavidin-modified agarose beads. After repeated washing, the enriched probe-binding proteins were eluted, separated by SDS‒PAGE, and detected by silver staining. As shown in Fig. 2A, one major band representing an approximately 70 kD protein was observed in the **S1g-2-probe** lane but not in the **S1-probe** lane or **S1n-probe** lane. Western blot experiments confirmed that the 70-kD protein was the Hsp70 protein, which is consistent with the binding target of **S1g-2** in vitro*,* while the **S1-probe** pulled down high amounts of Bcl-2 and Mcl-1, of which a nominal amount was pulled down by the **S1g-2-probe** or **S1n-probe**. **S1g-2-probe** enriched Hsp70 could be completed in a concentration-dependent manner with **S1g-2**, while **S1-probe** enriched Bcl-2 and Mcl-1 could be completed with **S1** (Additional file 1: Fig. S2B). These results suggested that Hsp70 is the autophagy-inhibiting target of **S1g-2**, while Bcl-2/Mcl-1 is the target of **S1** in living cells.

We next investigated whether Hsp70-Bim PPI disruption is the autophagy-inhibiting mechanism underlying the effect of **S1g-2**. We evaluated the dose-dependent disruption of the Hsp70-Bim PPI by **S1g-2** in parallel with autophagy inhibition. Through co-immunoprecipitation (co-IP) experiments, we found that the amount of Bim that coimmunoprecipitated with Hsp70 was increased by approximately 2–threefold in HEK293T cells after the induction of HBSS, H_2_O_2_ or VP-16 stress (Fig. 2B and Additional file 1: Fig. S3A). **S1g-2** at concentrations of 5 and 10 μM led to a dose-dependent disruption of the Hsp70-Bim PPI, an effect that was paralleled by a dose-dependent decrease in the LC3-II/LC3-I ratio and an increase in the p62 level (Fig. 2B and Additional file 1: Fig. S3A). We also measured the amount of Beclin 1 in complex with Bcl-2 or Hsp70. As shown in Additional file 1: Fig. S3B, **S1** induced the release of Beclin 1 from Bcl-2, as reported previously [18]. **S1** might also have released Beclin 1 from Mcl-1, the other cellular target of **S1** and one guard protein of Beclin 1. **S1g-2** disrupted the Hsp70-Bim PPI, while it had little effect on Beclin 1 that was associated with either Hsp70 or Bcl-2, ruling out the possibility that Beclin 1 is involved in the mechanism underlying the Hsp70-Bim PPI. Given the previously revealed autophagy-inhibitory Beclin 1-Bim PPI [22], we measured the Bim level in Beclin 1 immunoprecipitates. Consistent with previous reports, HBSS induced a decrease in the level of Bim associated with Beclin 1 [22]. Neither **S1g-2** nor **S1** affected the Beclin 1-Bim PPI, consistent with the finding that the region in Bim critical for Beclin 1 binding differs from that needed for the Bcl-2 and Hsp70 PPI (Additional file 1: Fig. S3B).

These findings prompted us to validate the dependency of the Hsp70-Bim PPI on autophagy inhibition mediated by **S1g-2** through cell-based shRNA assays. We found that Hsp70 or Bim knockdown led to a significant decrease in the level of HBSS-induced autophagy (Fig. 2C). Moreover, the effect of **S1g-2** on autophagy inhibition was abolished by either Hsp70 or Bim knockdown (Fig. 2C).

To examine whether basal autophagy is regulated by the Hsp70-Bim PPI, we exposed HEK293T cells directly to each of the three compounds, and then, the levels of LC3 and p62 were measured by Western blotting. The lysosome inhibitor bafilomycin A1 (BafA1), which is widely used to reveal basal autophagy, was used as a control, and the results showed a significant increase in the LC3-II/LC3-I ratio and p62 level (Fig. 2D). **S1** promoted basal autophagy, as shown by the increase in the LC3-II/LC3-I ratio and the decrease in p62 level, consistent with the inhibitory effect of Bcl-2 on basal autophagy. In contrast, **S1g-2** exerted little effect on the LC3-II/LC3-I ratio or p62 level, indicating that basal autophagy proceeds mostly independently of the Hsp70-Bim PPI (Fig. 2D). We also compared the Hsp70-Bim PPI requirement for basal and starvation-induced autophagy by treating cells that had been starved in HBSS medium or incubated with BafA1-supplemented medium with **S1g-2**. We found that the change in the LC3-II/LC3-I ratio induced by starvation was exacerbated by **S1g-2** treatment (2.8 vs. 6.3), but negligible changes were found to the cells treated with BafA1 (Fig. 2E). The results showed that in the absence of the Hsp70-Bim PPI, stress-induced autophagy was impaired, whereas basal autophagy was largely unaffected.

Taken together, these results demonstrated that the Hsp70-Bim PPI is the target critical for the autophagy-inhibiting activity of **S1g-2**. This finding highlights that **S1g-2** is an ideal tool to study the previously unknown molecular biological roles of the Hsp70-Bim PPI in autophagy process.

### Hsp70-Bim PPI regulates mitophagy

We previously utilized the immunoprecipitation–mass spectrometry (IP–MS) approach to identify cellular proteins associated with the Hsp70-Bim PPI [12]. Given the role of the Hsp70-Bim PPI in modulating autophagy as indicated in the aforementioned experiments, we reanalyzed the IP–MS data with a focus on the expression of autophagy-related proteins, among which that of parkin and TOMM20, two mitophagy-regulated proteins, showed the highest fold change. This analysis suggested that disruption of the Hsp70-Bim PPI by **S1g-2** may exert a selective effect on mitophagy.

**Fig. 3:**
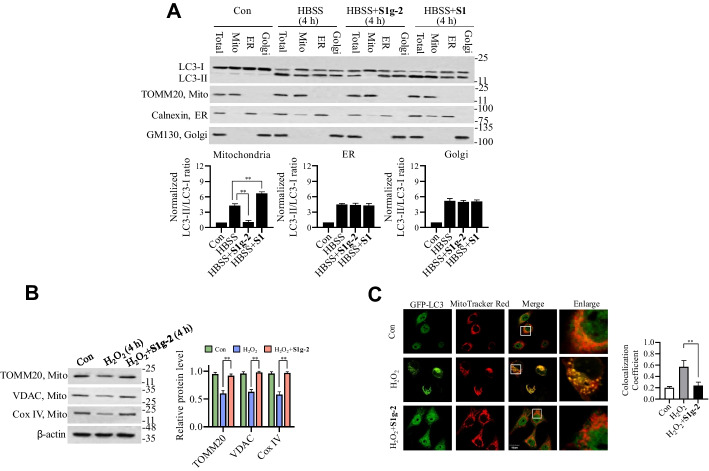
Disruption of Hsp70-Bim PPI by **S1g-2** inhibits mitophagy in response to various stresses. **A** Western blots analysis of LC3 level in isolated mitochondria, ER or Golgi from HEK293T cells treated with HBSS in the presence or absence of 10 μM **S1g-2** for 4 h. An equivalent of DMSO were added to the compound untreated group as vehicle control. Western blot of subcellular fraction probed with antibodies specific for organelle-specific marker proteins: mitochondria (TOMM20), ER (Calnexin), Golgi (GM130). The graphs show (mean ± SD, *n* = 3 biologically independent experiments) LC3-II/LC3-I ratios of each treatment normalized to the LC3-II/LC3-I ratio of control cells. **P < 0.01 (one-way ANOVA test). **B** Western blot analysis of TOMM20, Calnexin and GM130 in HEK293T cells treated with H_2_O_2_ in the presence or absence of 10 μM **S1g-2** for 4 h. An equivalent of DMSO were added to the compound untreated group as vehicle control. Right panel: relative levels of TOMM20, Calnexin and GM130 normalized to β-actin. The data are expressed as the mean ± SD (*n* = 3 biologically independent experiments). **P < 0.01 (one-way ANOVA test). **C** Representative colocalization images of GFP-LC3 (green) and mitochondria (MitoTracker Red). Quantification of the colocalization coefficient between GFP-LC3 and MitoTracker Red displayed as Pearson coefficients in the colocalized volume (1, perfect correlation; 0, no correlation). The data are expressed as the mean ± SD (*n* = 3 biologically independent experiments). **P < 0.01 (one-way ANOVA test), *n* = 5–6 dishes, 20 fields per dish. All figures represent the results from *n* = 3 biologically independent experiments

To test this hypothesis, we evaluated the effect of **S1g-2** on autophagy in specific organelles after stimulation by isolating mitochondria, endoplasmic reticulum (ER) and Golgi, followed by Western blot analysis of the LC3 levels. The purity and amount of isolated organelles used for the LC3 analysis were confirmed by immunoblotting with antibodies against organelle-specific proteins (Fig. 3A). **S1** was assayed in parallel with the control. As shown in Fig. 3A, HBSS induced an increase in the LC3-II/LC3-I ratio in mitochondria, the ER and the Golgi apparatus, consistent with previous reports on starvation-induced autophagy involving multiple organelles [23]. **S1g-2** significantly decreased the LC3-II/LC3-I ratio in the mitochondrial fraction but not in the ER or Golgi fraction. In contrast, **S1** induced a further increase in the LC3-II/LC3-I ratio in mitochondria but not in the ER or Golgi fraction. These findings were consistent with a previous report on another Bcl-2 inhibitor, ABT-737, which stimulates mitophagy but not reticulophagy [24]. Additionally, we measured ER protein levels in isolated mitochondria undergoing mitophagy, which are indicative of the colocalization of mitochondria with ER during mitophagy, which has been reported in previous studies [25]. The results showed a selective effect of **S1g-2** on the LC3-II/LC3-I ratio in mitochondria but not in the ER, suggesting that **S1g-2** specifically inhibited mitophagy. Similarly, the H_2_O_2_- or VP-16-induced increase in the LC3-II/LC3-I ratio in mitochondria was attenuated by **S1g-2** (Additional file 1: Fig. S4).

To further characterize the role of Hsp70-Bim in mitophagy, we evaluated the effect of **S1g-2** on oxidative-induced mitophagy via other assays. Mitophagy was identified by the Western blot analysis of various mitochondrial proteins and by measuring LC3 and MitoTracker colocalization with a confocal microscope. The results showed that **S1g-2** abolished the loss of the mitochondrial proteins TOMM20, VDAC and Cox IV that had been induced by H_2_O_2_ treatment (Fig. 3B), supporting the conclusion that disruption of the Hsp70-Bim PPI impairs mitophagy.

Next, we visually examined the colocalization of LC3 with mitochondria after **S1g-2** treatment. HEK293T cells overexpressing GFP-LC3 were established. To quantify LC3 redistribution, Pearson’s correlation coefficient (0: no correlation/colocalization, 1: complete correlation/colocalization) between GFP-LC3 and MitoTracker Red was determined. Using confocal microscopy, we saw that H_2_O_2_ induced a significant increase in the Pearson’s correlation (0.57 ± 0.15) (Fig. 3C), indicating the formation of GFP-LC3 puncta in mitochondria. **S1g-2** significantly decreased the degree of overlapping localization (0.24 ± 0.10), confirming the role played by the Hsp70-Bim PPI in mitophagy.

### The Hsp70-Bim PPI recruits TOMM20 and parkin to facilitate ubiquitination

**Fig. 4:**
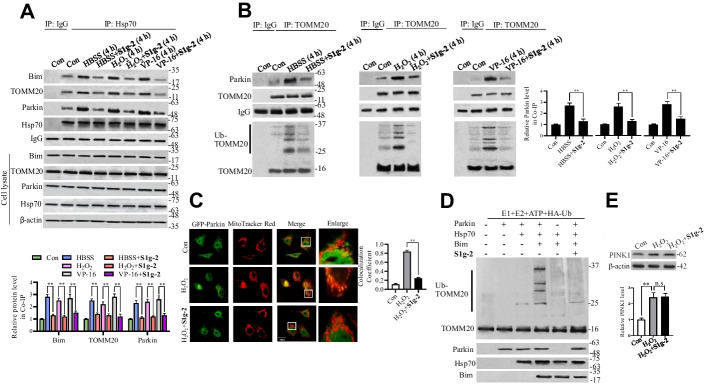
**S1g-2** inhibits the association between TOMM20 and parkin followed by parkin-mediated TOMM20 ubiquitination. **A** Co-IP analysis of Hsp70 interactions with Bim, TOMM20 and parkin in HEK293T cells treated with 0.5 mM H_2_O_2_, 34 μM VP-16 or HBSS in the presence or absence of 10 μM **S1g-2** for 4 h. An equivalent of DMSO were added to the compound untreated group as vehicle control. **P < 0.01 (one-way ANOVA test). **B** Co-IP analysis of TOMM20 interactions with parkin in HEK293T cells treated as described in **A**. **P < 0.01 (one-way ANOVA test). Ubiquitinated TOMM20 was visualized by Western blot analysis using anti-ubiquitin. **C** Representative colocalization images of GFP-parkin expressed HeLa cells treated with H_2_O_2_ in the presence or absence of 10 μM **S1g-2** for 4 h. Cells were stained for mitochondria (MitoTracker Red). Quantification of the colocalization coefficient between GFP-parkin and MitoTracker Red displayed as Pearson coefficients in the colocalized volume. The data are expressed as the mean ± SD (*n* = 3 biologically independent experiments). **P < 0.01 (one-way ANOVA test), *n* = 5–6 dishes, 20 fields per dish. **D** In vitro ubiquitination assay of TOMM20 in the presence or absence of Hsp70, Bim and **S1g-2** respectively or in combination. All figures represent the results from *n* = 3 biologically independent experiments. **E** Western blot analysis of PINK1 in HEK293T cells treated with H_2_O_2_ in the presence or absence of 10 μM **S1g-2** for 4 h. The graphs show relative levels of PINK1 normalized to β-actin. The data are expressed as the mean ± SD (*n* = 3 biologically independent experiments). **P < 0.01 (one-way ANOVA test); n.s. indicates no significance

To explore the mechanism by which the Hsp70-Bim PPI mediates stress-induced mitophagy, we examined the influence of **S1g-2** on the biological function of TOMM20 and parkin. We first evaluated whether the Hsp70-Bim PPI affects the TOMM20 and parkin association. Through a co-IP assay, we found that upon H_2_O_2_, HBSS or VP-16 treatment, Bim, TOMM20 and parkin were increasingly recruited to Hsp70, as indicated by their immunoprecipitation levels (Fig. 4A). Moreover, an increased association between parkin and TOMM20 was observed (Fig. 4B). **S1g-2** decreased the amounts of Bim, TOMM20 and parkin pulled down by Hsp70 immunoprecipitation, and the parkin association with TOMM20 was decreased (Fig. 4A and B), suggesting that parkin–TOMM20 association depends on the Hsp70-Bim PPI.

Parkin is an E3 ubiquitin ligase that is translocated to mitochondria, where it ubiquitinates mitochondrial outer membrane substrates, including TOMM20, and subsequently binds autophagy receptor proteins. As expected, confocal microscopy imaging revealed that **S1g-2** interfered with the colocation of GFP-parkin with mitochondria in HeLa cells following H_2_O_2_ treatment (the Pearson’s correlation was 0.21 ± 0.11 vs. 0.82 ± 0.17) (Fig. 4C). Stress-induced parkin translocation was found to be inhibited by either Bim or Hsp70 knockdown (Additional file 1: Fig. S5). These data demonstrated that the Hsp70-Bim PPI is a determinant of parkin translocation upstream of mitophagy pathways activated by different types of stress.

Furthermore, we established that the Hsp70-Bim PPI promotes parkin-mediated TOMM20 ubiquitination. First, **S1g-2** significantly decreased the level of TOMM20 ubiquitination in parallel with the disassociation of parkin and TOMM20, as shown in Fig. 4B. Second, we found that depletion of either Bim or Hsp70 by shRNA decreased the parkin association with TOMM20 as well as TOMM20 ubiquitination induced by HBSS (Additional file 1: Fig. S6A). Third, we immunoprecipitated parkin from HEK293T cell lysates and examined its E3 ubiquitin ligase activity in the presence and absence of Hsp70 and Bim in vitro. Immunoprecipitated parkin showed weak ubiquitination of TOMM20 in the presence of E1 and E2 (Fig. 4D). When Hsp70 was included in the reaction system, the level of TOMM20 ubiquitination was unchanged, but in the presence of the recombinant BimEL protein at a molar ratio of 1:10, the amount of ubiquitinated TOMM20 was significantly increased. The addition of **S1g-2** completely abolished TOMM20 ubiquitination induced by parkin. Taken together, we concluded that the Hsp70-Bim PPI recruits parkin and TOMM20 together, resulting in TOMM20 ubiquitination.

AMP-activated protein kinase (AMPK), an energy sensor, is activated by various stresses and stimulates autophagy mediated by ULK1. To evaluate the influence of the Hsp70-Bim PPI on cellular energy balance, we evaluated the effect of **S1g-2** on AMPK activation. As shown in Additional file 1: Fig. S6B, HBSS treatment induced an increase in AMPK phosphorylation due to the low ATP/AMP ratio, while **S1g-2** exerted no effect on the AMPK activation level, together indicating an energy-independent mechanism.

PTEN-induced kinase 1 (PINK1), a protein kinase, has been identified as a client of Hsc70/Hsp70 [26]. In response to mitophagy stress, PINK1 accumulates on mitochondria, and it recruits parkin from the cytosol to mitochondria. To characterize the role played by Hsp70-Bim in PINK1, we evaluated the effect of **S1g-2** on the PINK1 protein level. Consistent with previous reports, the PINK1 level was increased after mitochondria were damaged [26], while **S1g-2** exerted no effect on the PINK1 expression level (Fig. 4E), showing that the mechanism of the Hsp70-Bim PPI is not through regulating the stability of PINK1.

### Bax and Bak are dispensable for Hsp70-Bim PPI-mediated mitophagy

**Fig. 5:**
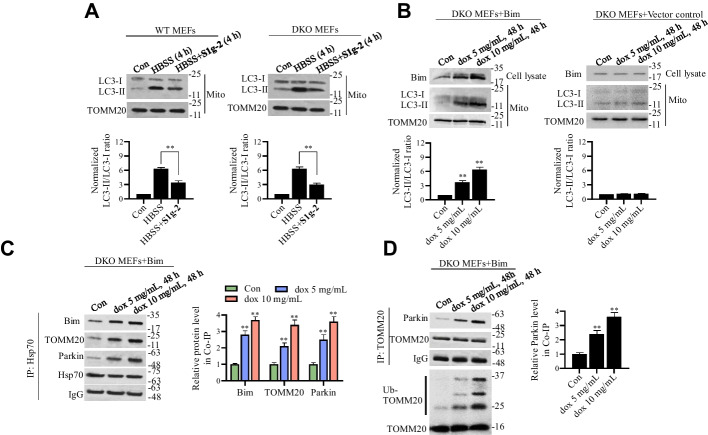
Hsp70-Bim PPI induced mitophagy independent on Bax and Bak. **A** Western blot analysis of LC3 level in isolated mitochondria from MEFs and *Bax/Bak* DKO MEFs treated with HBSS in the presence or absence of 10 μM **S1g-2** for 4 h. An equivalent of DMSO were added to the compound untreated group as vehicle control. **P < 0.01 (one-way ANOVA test). **B** *Bax/Bak* DKO MEFs transfected with Bim or the respective vector control was treated with 5 mg/mL and 10 mg/mL dox respectively for 48 h, and then the cell lysates were applied for Western blot analysis of Bim. The harvested cells were applied for mitochondria isolation and the LC3 level was analyzed by Western blotting. **P < 0.01 (one-way ANOVA test). **C** Co-IP analysis of Hsp70 interactions with Bim, TOMM20 and parkin in *Bax/Bak* DKO MEFs treated as described in **B**. **P < 0.01 (one-way ANOVA test). **D** Co-IP analysis of TOMM20 interactions with parkin and TOMM20 ubiquitination which was visualized by Western blot analysis using anti-ubiquitin in *Bax/Bak* DKO MEFs treated as described in **B**. All figures represent the results from *n* = 3 biologically independent experiments. **P < 0.01 (one-way ANOVA test)

Because Bim is an apoptosis activator that directly binds and activates Bax/Bak [27], we evaluated whether Hsp70-Bim PPI-induced mitophagy is affected by apoptosis. We treated *Bax/Bak* double-knockout (DKO) mouse embryonic fibroblasts (MEFs) with HBSS alone or in combination with **S1g-2** and then performed Western blot analysis to measure LC3 levels. As shown in Fig. 5A, HBSS induced a significant increase in the LC3-II/LC3-I ratio in the mitochondria of both the MEFs and *Bax/Bak* DKO MEFs. **S1g-2** significantly decreased the level of LC3-II in *Bax/Bak* DKO MEFs and in MEFs (Fig. 5A). The data showed that the Hsp70-Bim PPI regulates mitophagy via a Bax/Bak-independent mechanism.

Next, we examined the role of Bim in mitophagy without interference with the Bim-killing capability. We stably expressed doxycycline-inducible Bim in *Bax/Bak* DKO MEFs, in which mitochondrion-mediated cell death pathways were inactivated, and evaluated the effect of Bim on mitophagy. We found that Bim induced mitophagy (Fig. 5B), increased the parkin and TOMM20 levels in Hsp70 immunoprecipitates (Fig. 5C), and enhanced the parkin-TOMM20 association and TOMM20 ubiquitination in a dose-dependent manner (Fig. 5D). These results revealed a novel mechanism by which Bim promotes mitophagy.

### Hsp70-Bim PPI disruption enhances apoptosis by inhibiting mitophagy

**Fig. 6:**
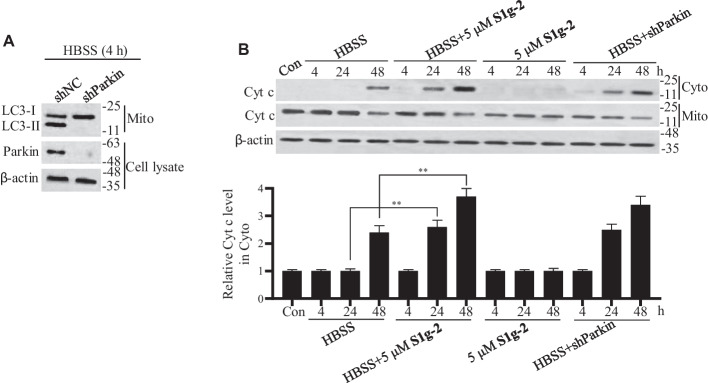
**S1g-2** or parkin shRNA enhanced cytochrome c release induced by HBSS.** A** Western blot analysis of the LC3 level in isolated mitochondria from NS shRNA-transfected or parkin shRNA-transfected HEK293T treated with HBSS. **B** Western blot analysis of cytochrome c in isolated mitochondria and cytosol supernatant from HEK293T cells or parkin shRNA-transfected cells after treatment with HBSS and 5 μM **S1g-2** alone or in combination for 24 and 48 h respectively. An equivalent of DMSO was added to the compound untreated group as vehicle control. All figures represent the results from *n* = 3 biologically independent experiments. **P < 0.01 (two-way ANOVA test)

The apoptosis-promoting effect of **S1g-2** administered at a higher dose than that used in this study to induce an Hsp70-Bim PPI blockade of the anti-apoptotic signaling pathway induced by Hsp70 clients (IC_50_ = 22.5 μM for HEK293T cells) has been identified [12]. Because the mitophagy-promoting survival effect has been well established, we asked whether **S1g-2** induces apoptosis through mitophagy inhibition when added at a concentration insufficient to interfere with anti-apoptotic client activity or to release enough Bim to activate apoptosis. We evaluated the mitochondrial function in cells treated with HBSS, H_2_O_2_ or VP-16 for a prolonged duration (24 and 48 h) with or without **S1g-2** treatment. Parkin knockdown by shRNA abolished stress-induced mitophagy (Fig. 6A) and thus parkin shRNA transfected cells were used as controls. As shown in Fig. 6B and Additional file 1: Fig. S7, various stresses led to cytochrome c release, which was enhanced by **S1g-2** or parkin shRNA, although **S1g-2** treatment alone did not induce cytochrome c release. By performing Annexin V staining experiments, we found that the apoptosis rate induced by exposure to various stresses for 24 and 48 h was increased by **S1g-2** or parkin shRNA (Additional file 1: Fig. S8). This result highlights the significance of Hsp70-Bim as a connection between autophagy and apoptosis, which can be used facilitate the discovery of new cancer therapies.

## Discussion

**Fig. 7:**
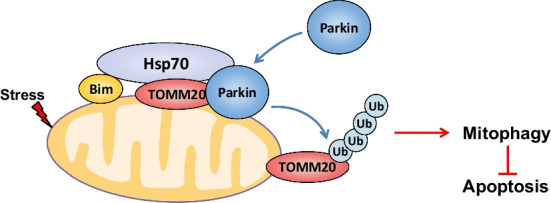
Proposed model for the role of Hsp70-Bim PPI in regulating mitophagy and apoptosis

Small-molecule inhibitors are useful tools for the elucidation of the mechanisms of cellular processes mediated by PPIs [28]. For example, ABT-737, representing BH3 mimetics, has been used to understand autophagy because it disrupts the Bcl-2/Beclin 1 PPI [24]. In the present study, analyses with **S1** and its analog **S1g-2**, which release Bim from Bcl-2 and Hsp70, respectively, revealed that the Hsp70-Bim PPI is a novel mitophagy factor that functions through a previously undiscovered molecular pathway (Fig. 7).

Hsp70-Bim exhibits specificity in mitophagy, which is a selective form of autophagy that mediates the turnover of mitochondria. Our findings demonstrated that Hsp70-Bim is dispensable for basal autophagy but determines induced mitophagy by recruiting parkin and TOMM20. Basal/constitutive autophagy—defined here as macroautophagy activity in cells cultured in normal medium supplemented with amino acids and serum—is a housekeeping mechanism [3]. The specificity of the Hsp70-Bim PPI in inducing mitophagy can be explained by the relatively low Hsp70-Bim PPI rate in unstimulated cells, in which parkin and TOMM20 were not recruited to mitochondria, as indicated by mitophagy induction following the Hsp70-Bim PPI rate increase in Bax/Bak DKO cells mediated by doxycycline-induced Bim expression. Since the specific autophagy is of greater therapeutic interest than basal autophagy and because mitophagy is often upregulated in therapy-resistant cancer cells and is a requirement for cancer stem cells (CSCs) [29, 30], the Hsp70-Bim PPI may be a more attractive therapeutic target than the general autophagy-related PPIs.

The specificity of the Hsp70-Bim PPI for inducing mitophagy further illustrated that **S1g-2** did not affect the activation of the universal regulator AMPK [31], suggesting that Hsp70-Bim PPI occurs at a point downstream of the mTOR-AMPK pathway. The essential role of AMPK in the T-cell response has been clearly described [32]. Hsp70-Bim bypasses, at least partially, the immune system to regulate autophagy, highlighting its significance as a selective therapeutic target that does interfere in the upstream factors linked with the immune system.

When the selectivity of the Hsp70-Bim PPI in mitophagy induction was demonstrated, Bim was identified as a more versatile player in maintaining mitochondrial function than had been previously appreciated. In addition to its role as an apoptosis activator, Bim promotes cell survival by inhibiting autophagy through its direct interaction with Beclin 1 [22]. Recently, an additional prosurvival function of Bim in cancer cells was discovered by our group, which showed that Bim functions as a cochaperone that facilitates Hsp70-induced stabilization of certain oncogenic clients [13]. In the present study, an addition prosurvival role for Bim in autophagy facilitation, independent of Beclin 1, was shown for the first time. The in vitro experiment involving TOMM20 ubiquitination revealed that Hsp70 combined with Bim, not Hsp70 itself, facilitated this outcome. The BH3 domain in Bim binds Hsp70 [13], but the Bim C-terminus binds Beclin-1 [22]. The multidomain binding sites in Bim and its multiple functions mediated via different autophagy regulators, namely, Hsp70 and Beclin 1, illustrates its versatile role.

It is reasonable to suggest that Bim plays its mitophagy-inducing role through conformation-dependent binding with Hsp70. In contrast to BAG4, Bim preferentially binds Hsp70 in an ADP-binding conformation, as we previously reported [13]. In contrast, BAG4 stabilizes the ATP-binding conformation of Hsp70. Therefore, Bim may promote a change in the Hsp70 conformation to recruit parkin, while BAG4 may drive Hsp70 to undergo the conformation that parkin disfavors, as suggested by diverse siRNA screening strategies [11]. Thus, we can conclude that the formation of a complex comprising Hsp70, Bim, parkin, and TOMM20 is formed via a change in the Hsp70 conformation after Hsp70 binding with Bim, as we have demonstrated that the Hsp70-Bim PPI is a conformation-related dimer [12–14]. The de novo contacts may preferentially promote TOMM20 involvement via a stable and cooperative organization with parkin to promote its ubiquitination.

In addition, our findings answered some questions raised by a study of ABT-737-induced autophagy to explain how Bcl-2 family proteins regulate parkin-mediated mitophagy. Martin’s laboratory reported that Bcl-2, Mcl-1 and other prosurvival members of the Bcl-2 family antagonized parkin-mediated mitophagy, whereas and Bcl-2 inhibitors enhanced it [33]. However, Martin’s group demonstrated that mitophagy inhibition by Bcl-2-like proteins was mediated independent of Beclin 1, raising the question, how do Bcl-2 family proteins impede parkin mitochondrial translocation? Our findings reveal a previously unidentified prosurvival mitophagy mechanism, in addition to that mediated by Beclin 1. Our discovery of the role played by the Hsp70-Bim PPI in facilitating parkin translocation, combined with our recent report on Bim transfer between Hsp70 and Bcl-2 [34], provides comprehensive insight into mitophagy regulation mediated by Bcl-2 family members. When Bim is released from the Bcl-2 protein by ABT-737, it binds to Hsp70, forming the Hsp70-Bim PPI, which promotes parkin-mediated mitophagy. The enhancing effect of **S1** on mitophagy in the present study supports this hypothesis.

From a practical point of view, active small molecules can be engineered into new drug candidates. A modulator that simultaneously directs both apoptosis and autophagy toward the same end, that is, cell death, is valuable because autophagy and apoptosis pathways sometimes lead to opposite cellular outcomes. Unfortunately, **S1** promoted cytoprotective mitophagy and exhibited anti-apoptotic functions, as shown in a previous study [18], as well as via the previous findings with ABT-737 experiments [35], indicating that it cannot induce apoptosis until the concentration is high enough to disrupt Bcl-2 complexes formed with cell-killing partners and overcome the cytoprotective mitophagy that it also induces. However, at relatively low concentrations, **S1g-2** indirectly induces apoptosis by inhibiting mitophagy; however, although released by **S1g-2**, the Bim level is insufficient to activate apoptosis or inhibit the oncogenic clients of Hsp70. We are not sure how mitophagy proceeds after apoptosis has been induced following an increase in **S1g-2** concentration; nonetheless, apoptosis induction is the ultimate goal of anticancer drugs, and **S1g-2** exhibits dual and continuous lethal activity within a wider dose window.

In addition, small molecules that block autophagy in a highly specific manner are urgently needed. **S1g-2** selectively regulates induced mitophagy since the basal level of Hsp70-Bim is much lower than that after stimulation. Compared with chloroquine, which modulates some central autophagy mechanisms and affects some autophagy-independent off-targets [36], **S1g-2** exhibits clean targeting in situ, as shown in this study. **S1g-2** may represent the next generation of autophagy modulator/apoptosis inducers, and its synergy with chemotherapies or targeted drugs that definitively induce autophagy is an attractive possibility.

Notably, the Hsp70-Bim PPI has been identified as an anti-CML target, and **S1g-2** exhibits potent activity against Bcr-Abl-independent TKI resistance [12]. This study also shows that **S1g-2** represents a new therapy avenue that kills two birds with one stone, since autophagy is an alternative therapeutic target in CML.

Our knowledge of the functions of a mitophagy modulator, the Hsp70-Bim PPI, and its link with oncogenic signaling must be increased in the future to produce more effective cancer treatments, including but not limited to analogs of **S1g-2**.

## Conclusion

Our findings demonstrate that the Hsp70-Bim PPI facilitates the formation of a complex involving parkin and TOMM20, which in turn facilitates parkin translocation to mitochondria, TOMM20 ubiquitination and mitophagic flux independent of Bax/Bak activity (Fig. 7).

## Supplementary Information

**Additional file 1. **Supplementary Figures.

## Data Availability

All data generated or analysed during this study are included in this published article.

## Abbreviations

Hsp70: Heat-shock protein 70
BH3: Bcl-2 homology 3
Bim: Bcl-2-interacting mediator of cell death
PPI: Protein–protein interaction
BafA1: Bafilomycin A1
CML: Chronic myeloid leukemia
VP-16: DNA damage agent etoposide
co-IP: Co-immunoprecipitation
TOMM20: Outer mitochondrial membrane 20
ER: Endoplasmic reticulum
AMPK: AMP-activated protein kinase
DKO: *Bax/Bak* Double-knockout
CSC: Cancer stem cells

## References

[1] He C, Klionsky DJ (2009). Regulation mechanisms and signaling pathways of autophagy. Annu Rev Genet.

[2] McConkey DJ (2017). The integrated stress response and proteotoxicity in cancer therapy. Biochem Biophys Res Commun.

[3] Antonucci L, Fagman JB, Kim JY, Todoric J, Gukovsky I, Mackey M, Ellisman MH, Karin M (2015). Basal autophagy maintains pancreatic acinar cell homeostasis and protein synthesis and prevents ER stress. Proc Natl Acad Sci U S A.

[4] Mizushima N (2005). The pleiotropic role of autophagy: from protein metabolism to bactericide. Cell Death Differ.

[5] Mele L, Del Vecchio V, Liccardo D, Prisco C, Schwerdtfeger M, Robinson N, Desiderio V, Tirino V, Papaccio G, La Noce M (2020). The role of autophagy in resistance to targeted therapies. Cancer Treat Rev.

[6] Lei Q, Tan J, Yi S, Wu N, Wang Y, Wu H (2018). Mitochonic acid 5 activates the MAPK–ERK–yap signaling pathways to protect mouse microglial BV-2 cells against TNFα-induced apoptosis via increased Bnip3-related mitophagy. Cell Mol Biol Lett.

[7] Das CK, Mandal M, Kögel D (2018). Pro-survival autophagy and cancer cell resistance to therapy. Cancer Metastasis Rev.

[8] Fimia GM, Kroemer G, Piacentini M (2013). Molecular mechanisms of selective autophagy. Cell Death Differ.

[9] Vara-Perez M, Felipe-Abrio B, Agostinis P (2019). Mitophagy in cancer: a tale of adaptation. Cells.

[10] Matsuda S, Nakanishi A, Minami A, Wada Y, Kitagishi Y (2015). Functions and characteristics of PINK1 and Parkin in cancer. Front Biosci (Landmark Ed).

[11] Hasson SA, Kane LA, Yamano K, Huang CH, Sliter DA, Buehler E, Wang C, Heman-Ackah SM, Hessa T, Guha R, Martin SE, Youle RJ (2013). High-content genome-wide RNAi screens identify regulators of parkin upstream of mitophagy. Nature.

[12] Song T, Guo Y, Xue Z, Guo Z, Wang Z, Lin D, Zhang H, Pan H, Zhang X, Yin F, Wang H, Uwituze LB, Zhang Z (2021). Small-molecule inhibitor targeting the Hsp70-Bim protein-protein interaction in CML cells overcomes BCR-ABL-independent TKI resistance. Leukemia.

[13] Guo Z, Song T, Wang Z, Lin D, Cao K, Liu P, Feng Y, Zhang X, Wang P, Yin F, Dai J, Zhou S, Zhang Z (2020). The chaperone Hsp70 is a BH3 receptor activated by the pro-apoptotic Bim to stabilize anti-apoptotic clients. J Biol Chem.

[14] Wang Z, Song T, Guo Z, Uwituze LB, Guo Y, Zhang H, Wang H, Zhang X, Pan H, Ji T, Yin F, Zhou S, Dai J, Zhang Z (2021). A novel Hsp70 inhibitor specifically targeting the cancer-related Hsp70-Bim protein-protein interaction. Eur J Med Chem.

[15] Levine B, Sinha SC, Kroemer G (2008). Bcl-2 family members: dual regulators of apoptosis and autophagy. Autophagy.

[16] Zhang Z, Song T, Zhang T, Gao J, Wu G, An L, Du G (2011). A novel BH3 mimetic S1 potently induces Bax/Bak-dependent apoptosis by targeting both Bcl-2 and Mcl-1. Int J Cancer.

[17] Zhang Z, Wu G, Xie F, Song T, Chang X (2011). 3-Thiomorpholin-8-oxo-8H- acenaphtho[1,2-b]pyrrole-9-carbonitrile (S1) based molecules as potent, dual inhibitors of B-cell lymphoma 2 (Bcl-2) and myeloid cell leukemia sequence 1 (Mcl-1): structure-based design and structure-activity relationship studies. J Med Chem.

[18] Zhong JT, Xu Y, Yi HW, Su J, Yu HM, Xiang XY, Li XN, Zhang ZC, Sun LK (2012). The BH3 mimetic S1 induces autophagy through ER stress and disruption of Bcl-2/Beclin 1 interaction in human glioma U251 cells. Cancer Lett.

[19] Liu WJ, Ye L, Huang WF, Guo LJ, Xu ZG, Wu HL, Yang C, Liu HF (2016). p62 links the autophagy pathway and the ubiqutin–proteasome system upon ubiquitinated protein degradation. Cell Mol Biol Lett.

[20] Lindqvist LM, Heinlein M, Huang DC, Vaux DL (2014). Prosurvival Bcl-2 family members affect autophagy only indirectly, by inhibiting Bax and Bak. Proc Natl Acad Sci U S A.

[21] Yang Y, Fiskus W, Yong B, Atadja P, Takahashi Y, Pandita TK, Wang HG, Bhalla KN (2013). Acetylated hsp70 and KAP1-mediated Vps34 SUMOylation is required for autophagosome creation in autophagy. Proc Natl Acad Sci U S A.

[22] Luo S, Garcia-Arencibia M, Zhao R, Puri C, Toh PP, Sadiq O, Rubinsztein DC (2012). Bim inhibits autophagy by recruiting Beclin 1 to microtubules. Mol Cell.

[23] Zhang Q, Kuang H, Chen C, Yan J, Do-Umehara HC, Liu XY, Dada L, Ridge KM, Chandel NS, Liu J (2015). The kinase Jnk2 promotes stress-induced mitophagy by targeting the small mitochondrial form of the tumor suppressor ARF for degradation. Nat Immunol.

[24] Maiuri MC, Criollo A, Tasdemir E, Vicencio JM, Tajeddine N, Hickman JA, Geneste O, Kroemer G (2007). BH3-only proteins and BH3 mimetics induce autophagy by competitively disrupting the interaction between Beclin 1 and Bcl-2/Bcl-X(L). Autophagy.

[25] Garofalo T, Matarrese P, Manganelli V, Marconi M, Tinari A, Gambardella L, Faggioni A, Misasi R, Sorice M, Malorni W (2016). Evidence for the involvement of lipid rafts localized at the ER-mitochondria associated membranes in autophagosome formation. Autophagy.

[26] Zheng Q, Huang C, Guo J, Tan J, Wang C, Tang B, Zhang H (2018). Hsp70 participates in PINK1-mediated mitophagy by regulating the stability of PINK1. Neurosci Lett.

[27] Kim H, Tu HC, Ren D, Takeuchi O, Jeffers JR, Zambetti GP, Hsieh JJ, Cheng EH (2009). Stepwise activation of BAX and BAK by tBID, BIM, and PUMA initiates mitochondrial apoptosis. Mol Cell.

[28] Arkin MR, Tang Y, Wells JA (2014). Small-molecule inhibitors of protein-protein interactions: progressing toward the reality. Chem Biol.

[29] Guan Y, Wang Y, Li B, Shen K, Li Q, Ni Y, Huang L (2021). Mitophagy in carcinogenesis, drug resistance and anticancer therapeutics. Cancer Cell Int.

[30] Naik PP, Birbrair A, Bhutia SK (2019). Mitophagy-driven metabolic switch reprograms stem cell fate. Cell Mol Life Sci.

[31] Li Y, Chen Y (2019). AMPK and autophagy. Adv Exp Med Biol.

[32] Tamás P, Hawley SA, Clarke RG, Mustard KJ, Green K, Hardie DG, Cantrell DA (2006). Regulation of the energy sensor AMP-activated protein kinase by antigen receptor and Ca2+ in T lymphocytes. J Exp Med.

[33] Hollville E, Carroll RG, Cullen SP, Martin SJ (2014). Bcl-2 family proteins participate in mitochondrial quality control by regulating Parkin/PINK1-dependent mitophagy. Mol Cell.

[34] Zhang H, Guo Z, Guo Y, Wang Z, Tang Y, Song T, Zhang Z (2021). Bim transfer between Bcl-2-like protein and Hsp70 underlines Bcl-2/Hsp70 crosstalk to regulate apoptosis. Biochem Pharmacol.

[35] Zinn RL, Gardner EE, Dobromilskaya I, Murphy S, Marchionni L, Hann CL, Rudin CM (2013). Combination treatment with ABT-737 and chloroquine in preclinical models of small cell lung cancer. Mol Cancer.

[36] Maycotte P, Aryal S, Cummings CT, Thorburn J, Morgan MJ, Thorburn A (2012). Chloroquine sensitizes breast cancer cells to chemotherapy independent of autophagy. Autophagy.

