# Supplementary material for: Hsp70-Bim interaction facilitates mitophagy by recruiting parkin and TOMM20 into a complex
Source: Cell Mol Biol Lett. 2023 May 26;28:46. doi: 10.1186/s11658-023-00458-5 (PMC10223935; doi:10.1186/s11658-023-00458-5)
Supplement: Supplementary file 1 — Additional file 1. Supplementary Figures. [file 11658_2023_458_MOESM1_ESM.docx]

**Hsp70-Bim interaction facilitates mitophagy by recruiting parkin and TOMM20 into a complex**

Ting Song^1,*^, Fangkui Yin^1^, Ziqian Wang^1^, Hong Zhang^2^, Peng Liu^2^, Yafei Guo^2^, Yao Tang^2^, and Zhichao Zhang^1,*^

^1^State Key Laboratory of Fine Chemicals, School of Chemical Engineering, Dalian University of Technology, Dalian, Liaoning (China)

^2^School of Life Science and Technology, Dalian University of Technology, Dalian, Liaoning (China)

^*^Correspondence to: Ting Song, Email: [songting@dlut.edu.cn](mailto:songting@dlut.edu.cn); Zhichao Zhang, Email: [zczhang@dlut.edu.cn](mailto:zczhang@dlut.edu.cn);

**Material and methods**

**Protein Expression and Purification**

Constructs of hHsp70 cDNA and a recombinant version of TOMM20 lacking the transmembrane regions [EMBO J (1997)16: 4267-4275] were subcloned into the pHis vector, and the resulting proteins with an N-terminal 6×His tag were produced in *E. coli* strain BL21 (DE3) containing the corresponding plasmid. Cells were produced at 37 °C to an optical density of 0.8 at 600 nm, induced by 0.5 mM IPTG at 37 °C for 5 hr, and then lysed by sonication and the lysate was cleared by centrifugation at 9000 rpm for 30 min, and proteins were purified from the soluble fraction using Ni-NTA resin (Qiagen), following the manufacturer’s instructions. The proteins were further purified by Sephadex G-75 size exclusion chromatography (GE Healthcare), before they were pooled and concentrated for *in vitro* ubiquitination assays.

**Cell viability assay**

Viability assessment in cells was examined by measuring the conversion of the tetrazolium salt (WST-8) to formazan according to the manufacturer’s instructions (CCK-8; Dojindo, Kumamoto, Japan). Briefly, cells (1.0 × 10^4^/well) were cultured and seeded into 96-well plates (three wells per group), and then the cells were treated with inhibitor in 48 hr. Approximately 20 μl of CCK-8 was added to the cells containing 200 μl medium, and the OD value of the cells was measured at 450 nm using a microplate reader (TECAN infinite F200 PRO, Mannedorf, Switzerland) according to the manufacturer’s instructions.

**Apoptosis assay**

Phosphatidylserine (PS) exposure was quantified by surface Annexin V-FITC staining. After treatment, cells were washed twice with phosphate-buffered saline (PBS) and incubated with a 1:40 solution of FITC-conjugated Annexin V for 10 min at room temperature. Stained cells were analyzed by flow cytometry. Analysis was performed using CellQuest Pro software (BD Biosciences).

**Doxycycline-inducible Bim expression vectors constructs**

pLVX-TetOne-EGFP-BCL2L11-Puro (Bim cDNA in the pLVX-Tet-On advanced vector) was purchased by MiaoLing Plasmid Sharing Platform (Hubei, Wuhan, China). Transfection initiated by pLVX-TetOne-EGFP-BCL2L11-Puro, PMD2.G and PSP-AX with 20 μL of Lipofectamine Reagent 2000 were added to HEK293T cultivated by DMEM media without serum for the amplification of plasmids. Media which containing the virus was collected and replace the fresh medium after 48 hours of transfection. The virus was collected 48 and 72 hours after changing the fresh medium. The collected cultured medium containing the virus was concentrated. Next, the concentrated virus was mixed with fresh medium (2:1) to infect *Bax/Bak* DKO MEF cells. The cells were cultured 30 days in a medium include 1.5 μg/mL of puromycin to select the transfectants until they are removed. Different concentrations of doxycycline (Beyotime, ST-039A) were used to induce the expression of Bim.

**Western blot**

After treatment the cells are collected centrifugalally, Wash the cells with buffer (PBS, phosphate-buffered saline; PH 7.4, 10 mM Na_2_HPO_4_, 140 mM NaCl, 1.8 mM KH_2_PO_4_, 2.7 mM KCl). Proteins were extracted from the cells by cell lysates buffer for Western and IP (Beyotime, P0013), normalize protein concentrations and then the proteins were separated by 12% SDS-PAGE (sodium dodecyl sulfate-polyacrylamide gel electrophoresis, 5% stacking gel and 12% separation gel). Then electrophoretically transferred the protein from the gel onto polyvinylidene difluoride (PVDF) blotting membrane (Millipore, IPVH00010). The membrane was blocked with 5% non-fat dry milk (Solarbio, D8340) in PBS. Primary antibody overnight incubation (4 °C, 12 hours), Wash 3 times with PBST, PBST buffer solution contains PBS solution and 0.1% Tween 20 (Solarbio, T8220), and 10 minutes each time. Incubate the secondary antibody at room temperature for 2 hours. Next, same as cleaning the primary antibody, PBST used to clean the PVDF membrane three times, and 10 minutes each time. Finally, after incubating for 2 minutes with Super Signal West Femto reagent (Thermo Fisher Scientific Inc.), perform exposure.

**Co-immunoprecipitation**

Collect (centrifuge at 1000 g, 4 °C for 5 min) and lyse (200 μL Cell lysis buffer for Western and IP, 4 degrees, 30 minutes) the cultured cells, then normalize protein concentrations, like sample preparation in Western blot. In order to prevent protein degradation, add 1% cocktail (Thermo Scientific, 1862209), EDTA (Thermo Scientific, 861217), PMSF (Beyotime, ST506-2). Respectively divide the sample into an input part (40 μL) and an immunoprecipitation part (160 μL). The input part is prepared like the Western blot sample, and the Ip part need to add 10μL gel beads (Protein A+G Agarose, Beyotime, P2012) then incubate at 4 degrees for 1 hour to remove the protein bound to the beads, next centrifuge to get the supernatant. Add the antibody that binds to the target protein to the supernatant and incubate overnight (12 hours) at 4 degrees. Add the agarose 40 μL again to decouple the antibody and the target protein bound to the antibody, then use PBS (containing 1% PMSF) to clean the agarose beads three times which bound to the antibody, target protein, and interaction with the target protein. Add the loading buffer to a boiling water bath for 15 minutes to separate the agarose beads from the protein. Finally, centrifuge and collect the supernatant. The following steps are the same as Western blot.


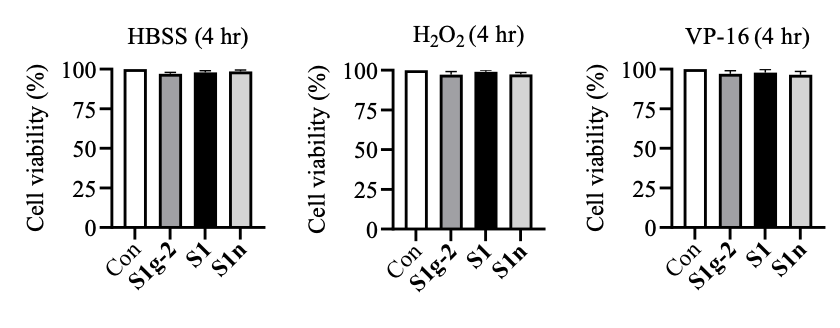


**Fig. S1.** No cell death was induced in HEK293T cells under various stress in the presence of compounds. HEK293T cells were treated with 0.5 mM H_2_O_2_, 34 μM VP-16 or cultured in HBSS in the presence of 10 μM **S1g-2**, 10 μM **S1** and 10 μM **S1n** respectively for 4 hr. The percentage of cell viability was determined by the CCK-8 assay. The data are expressed as the mean ± SD of n=3 biologically independent experiments.

**Fig. S2.** (**A**) Western blot analysis of the levels of LC3 and p62 in HEK293T cells treated with HBSS in the presence or absence of 10 μM **S1g-2**, 10 μM **S1** and 10 μM **S1n** respectively for 4 hr, using β-actin as a loading control. The graphs show (mean ± SD, n=3 biologically independent experiments) LC3-II/LC3-I ratios of each treatment normalized to the LC3-II/LC3-I ratio of control cells. ^**^*P*<0.01 (one-way ANOVA test). (**B**) Assays of the competitive binding of **S1g-2** to Hsp70/**S1g-2-probe**, **S1** to Mcl-1/**S1-probe**, and **S1** to Bcl-2/**S1-probe** enriched from HEK293T cells cultured in HBSS for 4 hr.

**Fig. S3. S1g-2** inhibits stress-induced autophagy by disrupting Hsp70-Bim PPI. (**A**) Co-IP analysis of Hsp70 interactions with Bim and Beclin 1 in HEK293T cells treated with 0.5 mM H_2_O_2_ or 34 μM VP-16 alone or in the presence of 5 μM **S1g-2**, 10 μM **S1g-2** and 10 μM **S1** respectively, and Western blot analysis of LC3 and p62 levels in cell lysates. The top graph shows (mean ± SD, n=3 biologically independent experiments) relative level of Bim and Beclin 1 in co-IP of each treatment normalized to that in control cells. The bottom graph shows (mean ± SD, n=3 biologically independent experiments) LC3-II/LC3-I ratios of each treatment normalized to the LC3-II/LC3-I ratio of control cells. ^*^*P*<0.05, ^**^*P*<0.01 (one-way ANOVA test). (**B**) Co-IP analysis of Beclin 1 interactions with Hsp70, Bcl-2 and Bim in HEK293T cells treated as in (**B**). All figures represent the results from n=3 biologically independent experiments.

**Fig. S4.** **S1g-2** inhibits mitophagy induced by either H_2_O_2_ or VP-16. Western blot analysis of LC3 level in isolated mitochondria from HEK293T cells treated with 0.5 mM H_2_O_2_, 34 μM VP-16 in the presence or absence of 10 μM **S1g-2** for 4 hr, using TOMM20 as the loading control. The graph shows (mean ± SD, n=3 biologically independent experiments) LC3-II/LC3-I ratios of each treatment normalized to the LC3-II/LC3-I ratio of control cells. ^**^*P*<0.01 (one-way ANOVA test).

**Fig. S5.** Either Bim or Hsp70 knockdown impaired Parkin translocation induced by H_2_O_2_. Western blot analysis of Parkin in isolated mitochondria from NS shRNA-transfected, Bim shRNA-transfected and Hsp70 shRNA-transfected HEK293T cells respectively with or without H_2_O_2_ treatment.

**Fig. S6.** Either Bim or Hsp70 knockdown impaired TOMM20 association with parkin and TOMM20 ubiquitination without influence on AMPK activation. (**A**) Co-IP analysis of TOMM20 interactions with parkin in NS shRNA-transfected, Bim shRNA-transfected and Hsp70 shRNA-transfected HEK293T cells respectively with HBSS treatment. Ubiquitinated TOMM20 was visualized by Western blot analysis using anti-ubiquitin. (**B**) Western blot analysis of p-AMPK (Thr172) and AMPK in HEK293T cells treated with HBSS in the presence or absence of 10 μM **S1g-2** for 4 hr.

**Fig. S7. S1g-2** or parkin shRNA enhanced cytochrome c release induced by H_2_O_2_ or VP-16. Western blot analysis of cytochrome c in isolated mitochondria and cytosol supernatant from HEK293T cells or parkin shRNA-transfected cells after treatment with 0.5 mM H_2_O_2_ or 34 μM VP-16 in the presence or absence of 5 μM **S1g-2** for 24 and 48 hr respectively. All figures represent the results from n=3 biologically independent experiments.

**Fig. S8.** HEK293T cells or parkin shRNA-transfected cells were treated with HBSS, 0.5 mM H_2_O_2_ or 34 μM VP-16 in the presence or absence of 5 μM **S1g-2** for 24 and 48 hr respectively and analyzed by flow cytometry for Annexin V positive cells. Data are shown as mean ± SD of 3 biologically independent experiments. ^**^*P*<0.01 (two-way ANOVA test).
